# Supplementary material for: L-Arabinose Alters the E. coli Transcriptome to Favor Biofilm Growth and Enhances Survival During Fluoroquinolone Stress
Source: Microorganisms. 2025 Jul 15;13(7):1665. doi: 10.3390/microorganisms13071665 (PMC12299780; doi:10.3390/microorganisms13071665)
Supplement: Supplementary file 1 [file microorganisms-13-01665-s001.zip › ArabinoseBiofilmSupplemental_v3.pdf]

Supporting Information

L-Arabinose alters the *E. coli* transcriptome to favor biofilm growth and enhances survival during fluoroquinolone stress

Katherine M. Austin, Jenna K. Frizzell, Audrey A. Neighmond, Isabella Moppel, Lisa M. Ryno*

§These authors contributed to this work equally
* To whom correspondence should be addressed:
e-mail: lryno@oberlin.edu
Telephone: 440-775-8238
Facsimile: 440-775-6682.

Table of Contents

Page

Figure S1:.. S3

Figure S2: S3

Figure S3: S4

Figure S4: S5

Figure S5: S6

Figure S6: S7

Figure S7: S8

Figure S8: S9

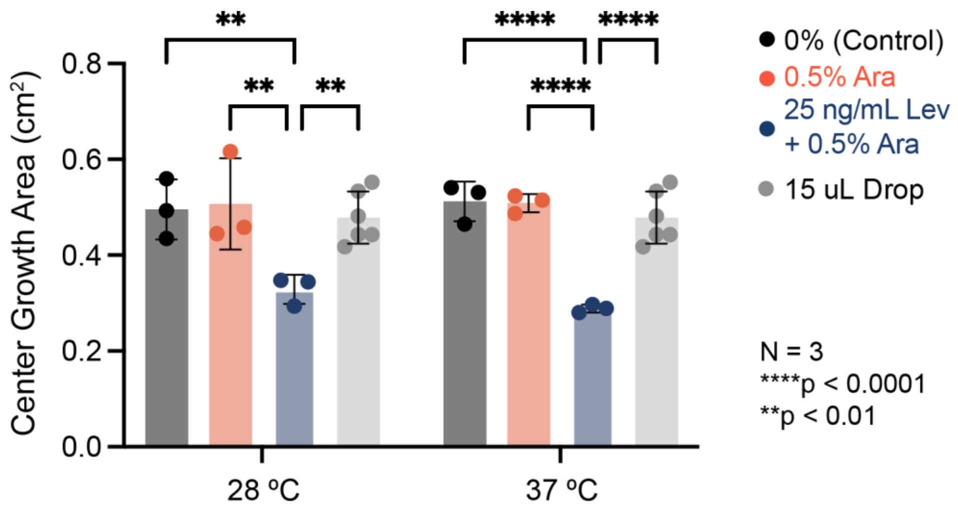

Figure S1. Area measurements of colony biofilms. Measurements completed using ImageJ. Statistics were determined using a two-way ANOVA with post-hoc Tukey test.

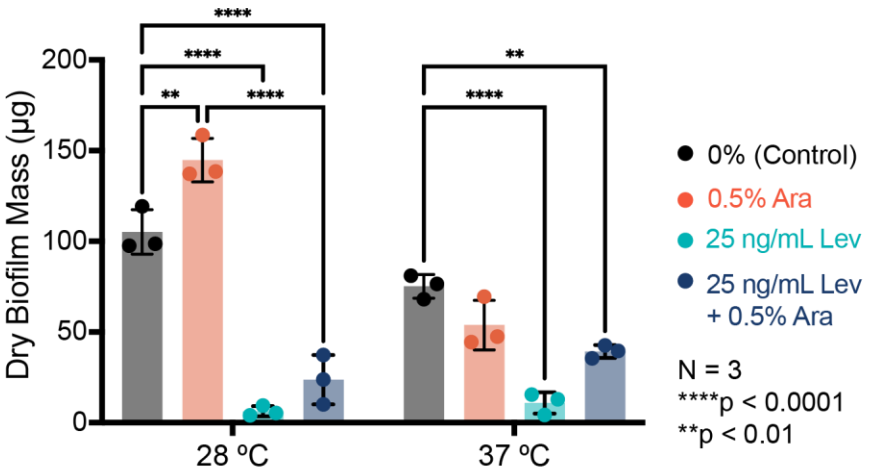

Figure S2. Mass measurements of dry biofilm from agar plate growth. Biofilm was grown on LB agar plates supplemented with either no added sugar (0%), 0.5% (*w/w*) L-arabinose, or 25 ng/mL levofloxacin. Statistics were determined using a two-way ANOVA with post-hoc Tukey test.

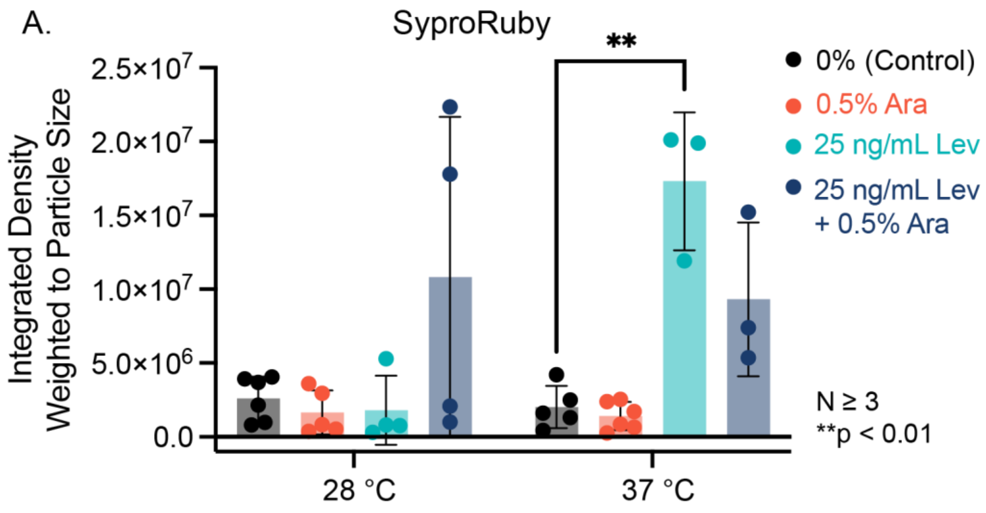

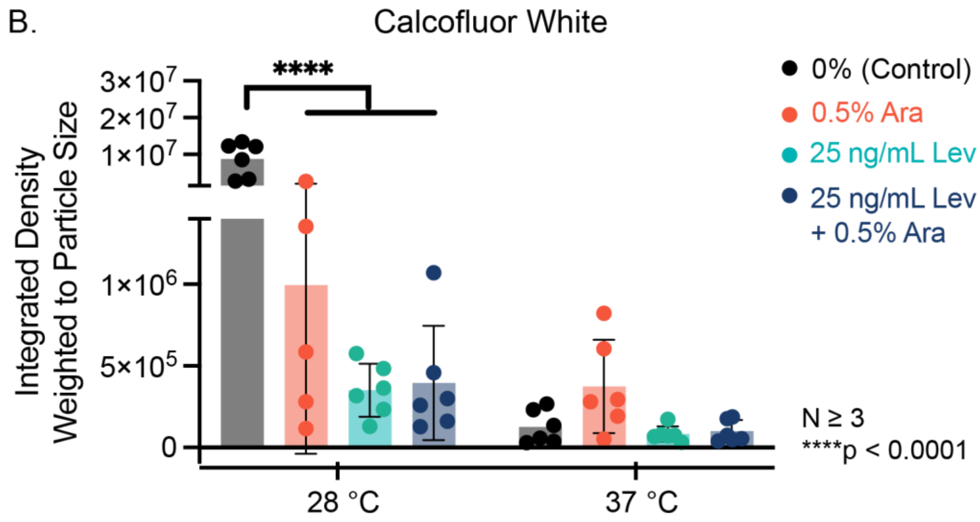

Figure S3. Biomolecule concentrations in the EPS quantified by confocal microscopy. **A.** Protein concentration of biofilms grown on glass wool for 48 h at 28 and 37 ºC quantified by SyproRuby stain. **B.** Carbohydrate concentration was also quantified using confocal microcopy of biofilms grown for 48 h on glass wool at 28 and 37 ºC and stained with Calcofluor White. Statistical significance determined using two-way ANOVA analysis with a post-hoc Tukey.

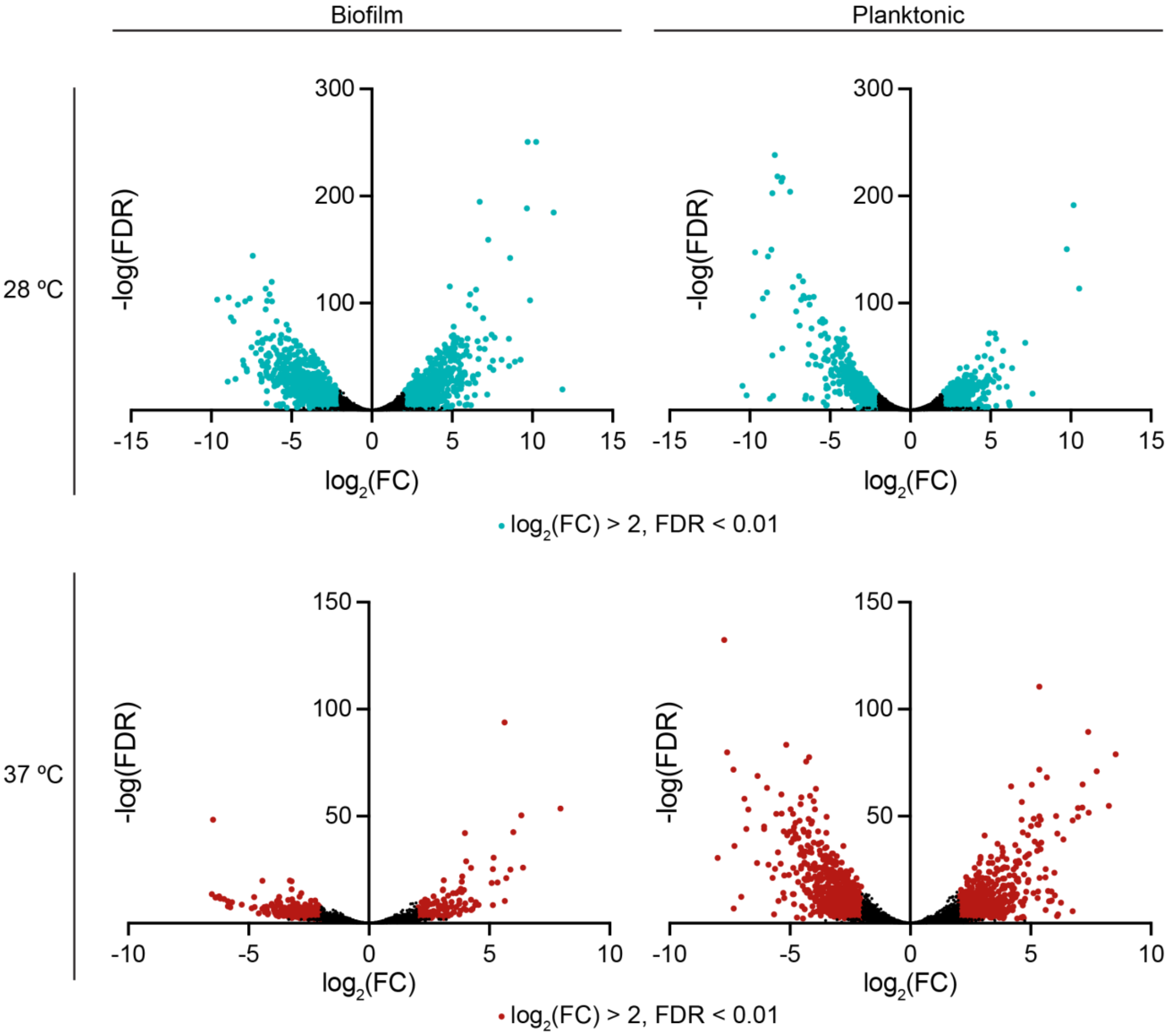

Figure S4. Volcano plots of RNA-seq data analyzed with DESeq2 for biofilm and planktonic cells harvested at 28 and 37 ºC. Color data (teal for 28 ºC, red for 37 ºC) indicate genes that are deemed “significant” in their differential expression, with $\log_2$(Fold Change) > 2 and false discovery rate < 0.01.

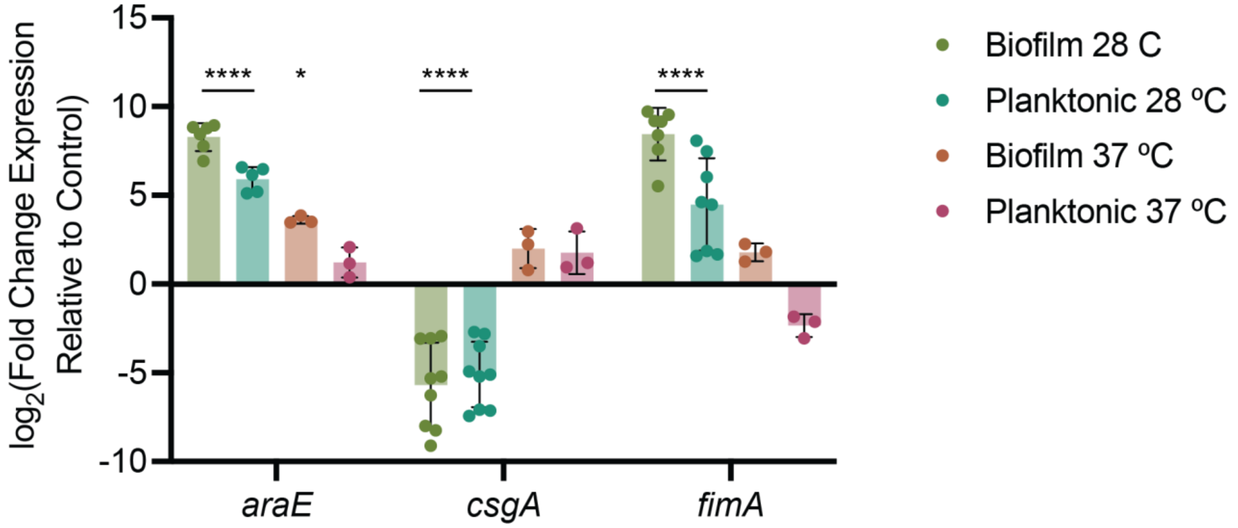

Figure S5. Gene expression data for select transcripts in rich and minimal media exposed to 0.5% (*w/w*) L-arabinose compared to control. Biofilms were grown on glass wool over 48 h at the temperatures indicated. Planktonic cells were harvested after 24 h growth. Grey dashed line indicates the comparative log2(fold expression) = 0 of control (0% sugar). Statistical significance determined using two-way ANOVA analysis with a post-hoc Tukey test in GraphPad Prism 10.

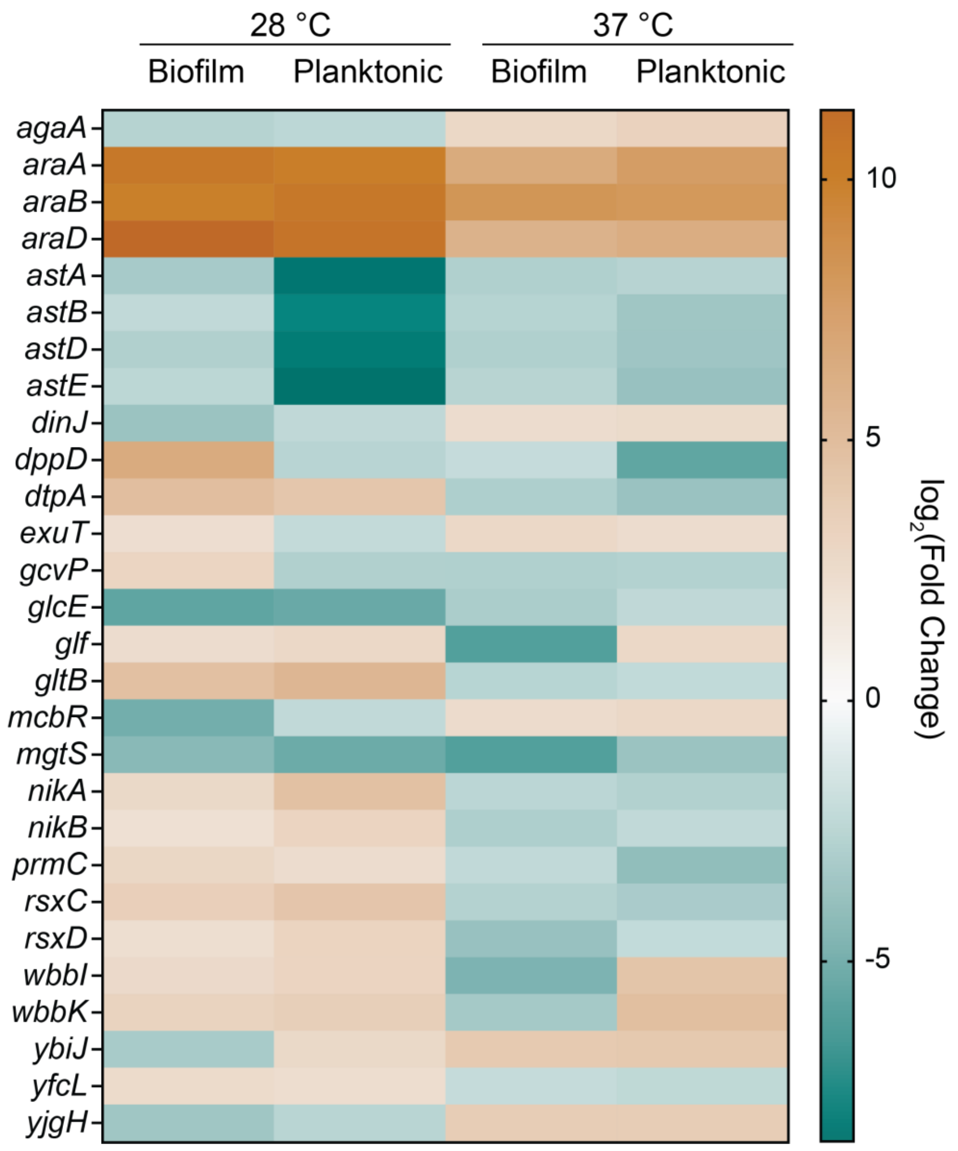

Figure S6. Heat map of significantly expressed genes (log_2(Fold Change) > 4, FDR < 0.01) found in all four experimental conditions.

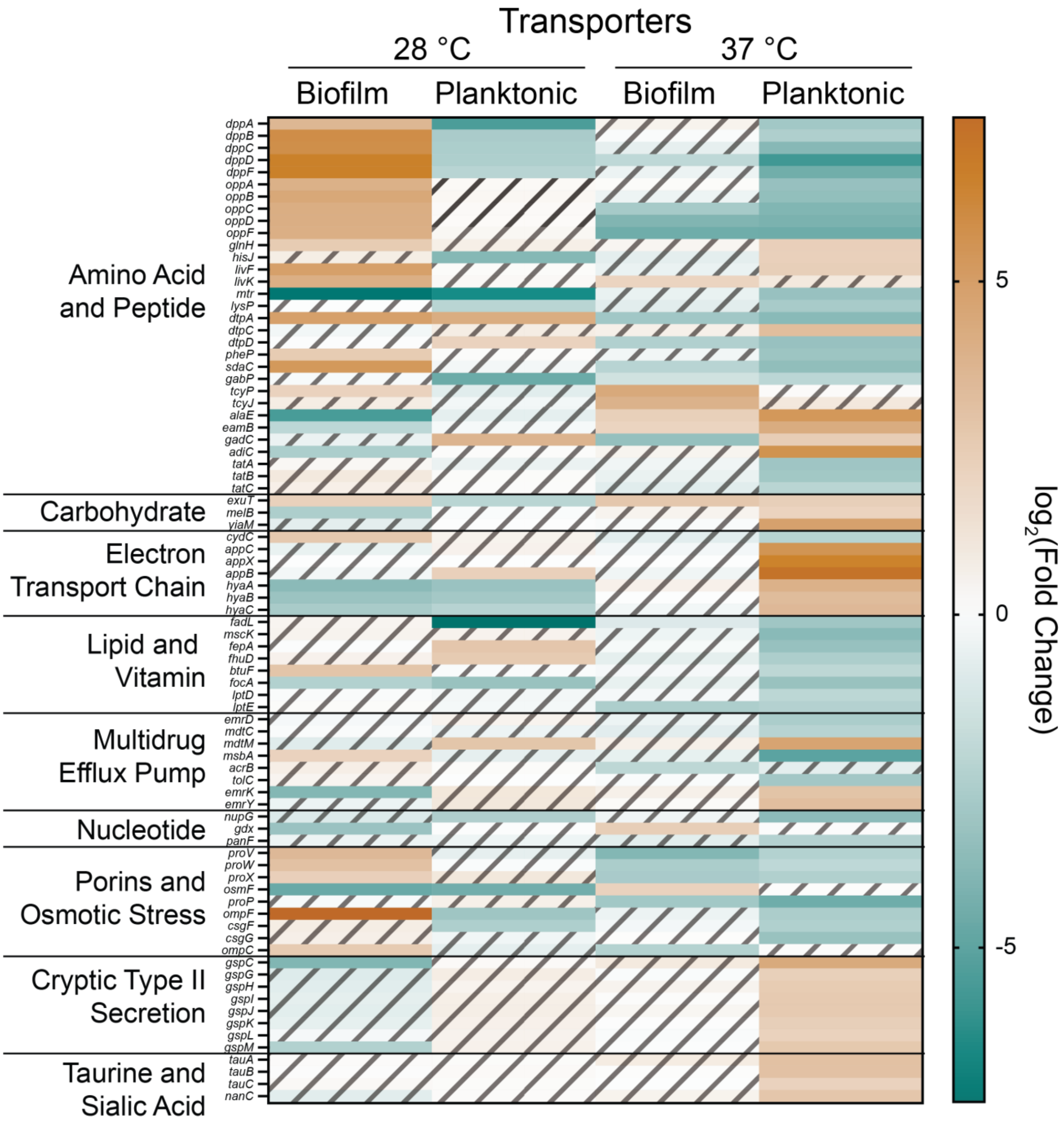

Figure S7. Heatmap of the $\log_2$(Fold Change) expression of the transporter genes found in an EcoCyc Omics analysis across all growth and media conditions. Hashed and faded cells represent genes that did not have significance as determined by our DESeq2 differential expression analysis cutoffs of $\log_2(FC) > 2$ and $p < 0.01$.

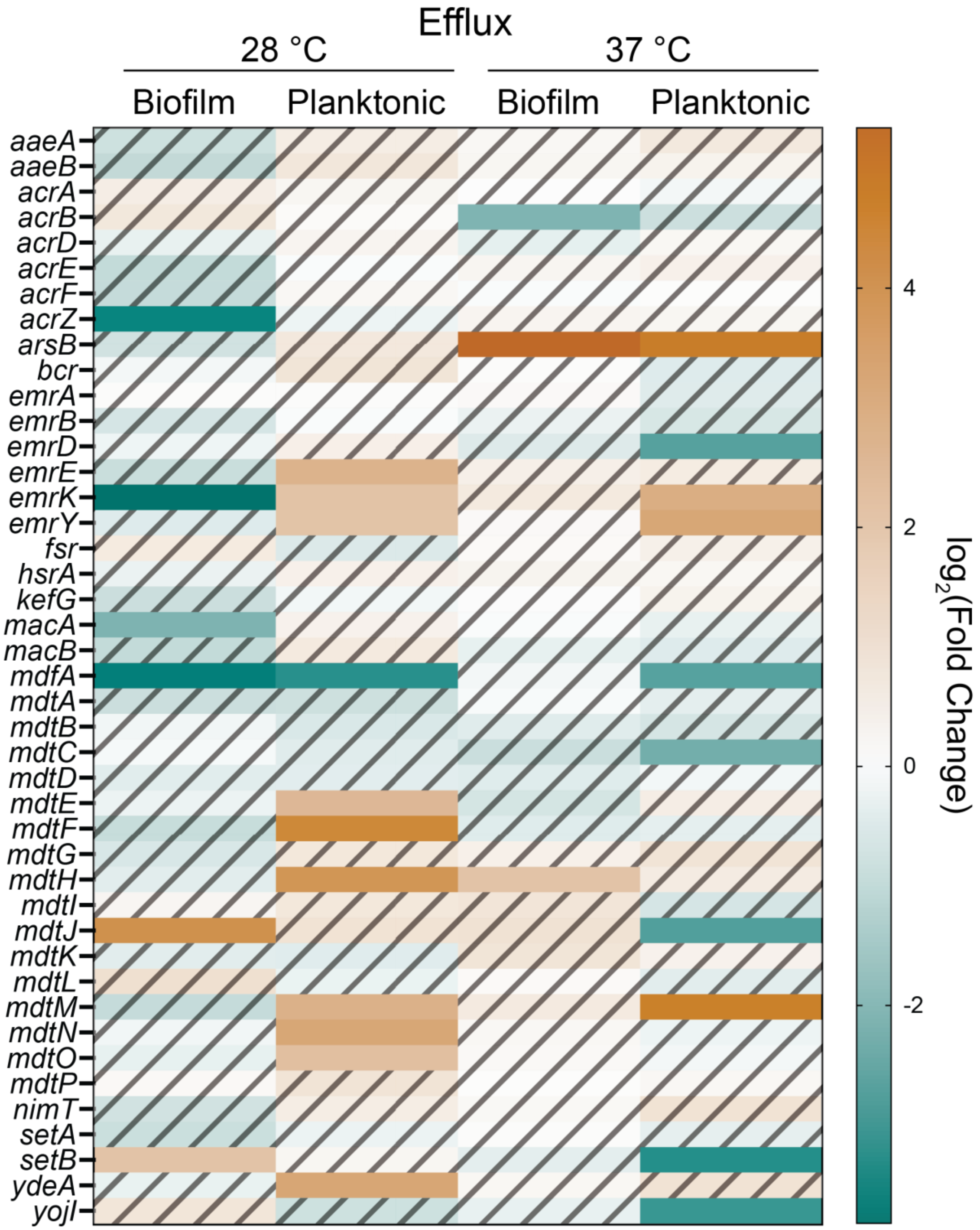

Figure S8. Heatmap of the $\log_2$(Fold Change) expression of efflux pumps identified in an EcoCyc Omics analysis for planktonic cells at all growth and media conditions. Hashed and faded cells represent genes that did not have significance as determined by our DESeq2 differential expression analysis cutoffs of $\log_2(FC) > 2$ and $p < 0.01$.
